# Incident of violence escalation of patients with psychiatric emergencies boarding in the emergency department in the central region of Thailand and its association: a prospective observational study

**DOI:** 10.1186/s12913-024-11228-0

**Published:** 2024-06-27

**Authors:** Aungsana Khlaisuk, Acharaporn Seeherunwong, Ketsarin Utriyaprasit, Autchariya Poungkaew, Sasima Tongsai

**Affiliations:** 1 Faculty of Nursing, Mahidol University, Bangkok, Thailand; 2 Department of Mental Health and Psychiatric Nursing, Faculty of Nursing, Mahidol University, Bangkok, Thailand; 3 Department of Surgical Nursing, Faculty of Nursing, Mahidol University, Bangkok, Thailand; 4 Department of Medical Nursing, Faculty of Nursing, Mahidol University, Bangkok, Thailand; 5 Research Department, Faculty of Medicine Siriraj Hospital, Mahidol University, Bangkok, Thailand

**Keywords:** Emergency department, Nurse competency, Psychiatric emergencies, Triage, Thailand, Violence escalation, Violent behavior

## Abstract

**Background:**

This study investigates the incidence of violence escalation among psychiatric emergency patients admitted to general emergency departments in hospitals in central Thailand. In addition, patient and service delivery system factors associated with the survival time of violence escalation in 16 emergency departments in the central region of Thailand are determined. This is a prospective observational study, and the study sample includes 507 psychiatric emergency patients who are ≥ 18 years old. The patients are selected through stratified random and purposive sampling.

**Methods:**

Patient data—including demographic data, emergency services used, and clinical characteristics—are analyzed using descriptive statistics. The Kaplan–Meier method estimates the violence escalation curve, and the log-rank test compares the violence escalation-free time between the levels of the violent behavior group. In addition, univariable and multivariable Cox proportional hazard analyses are performed to investigate the factors affecting violence escalation.

**Results:**

The incidence of violence escalation in psychiatric emergency patients in the emergency department is 7.3%, whereas the incidence rate of violence escalation is 3 per 100 psychiatric emergency patient visit hours. Factors affecting violence escalation include the violent behavior score at triage (aHR = 2.004; 95% CI: 1.051–3.823) and the nurse competency score (aHR = 0.147; 95% CI: 0.032–0.680).

**Conclusions:**

Assessing the violent behavior of psychiatric emergency patients at triage may assist emergency providers in monitoring patient behavior and providing early intervention to prevent the escalation of violent behavior. Furthermore, training emergency nurses in psychiatric emergency care is necessary.

**Supplementary Information:**

The online version contains supplementary material available at 10.1186/s12913-024-11228-0.

## Introduction

Patients’ violent behavior in hospital emergency rooms is severe and has a remarkable effect. The incidence of violence in the emergency department is strongly correlated with psychiatric patients [1, 2]. The study on violence incidents in psychiatric emergency patients visiting the general emergency department accounted for 3.4%–9% [3, 4]. Violence in the emergency department negatively impacts the provider and emergency service systems. In a previous study, violent incidents were correlated with negative emotions, negative physical symptoms [5], and intention to resign from the nursing job [6–8], which had a reversible effect on service systems and patient safety [9]. Psychiatric emergency patients must use services in the general emergency department [10, 11]; this trend is increasing [12–14]. In addition, the international organization [15] and national organization [16, 17] support policies for developing emergency psychiatric care services in general emergency departments. Therefore, improving psychiatric services in general emergency departments is necessary to address the increasing demand for care and prevent violence related to psychiatric patients.

Violence is a multifaceted issue in the emergency department and is influenced by several factors [18–20]. Herein, Holden’s framework of patient safety was applied—which proposes that patient safety results from the interaction among various factors in the work system and working processes [21]. The work system is person-centered and includes patients and providers. Patient characteristics are essential factors influencing violence [21]. Reportedly, violence is common among people who are young adults [1, 4, 19], have physical comorbidity, and present two or more psychiatric disorders [19]. Aggressive or threatening behavior during triage may indicate a potentially violent incident in the emergency room [9]. Provider factors include a lack of skills to manage violent behavior and the workload of emergency nurses [22]. In addition, a shortage of psychiatrists to care for psychiatric emergency patients may cause delays in psychiatric evaluations [22], resulting in violent behavior [23]. Safety training policies are also crucial for addressing violence [19]. Moreover, emergency rooms using clinical practice guidelines and the availability of restraint devices for managing violent behavior are related to violent incidents [24]. Overcrowding in the emergency room is a notable factor that influences violence in the emergency department [25–27]. However, a separate room for closely monitoring behavior increases safety [19]. Violent behavior is more common in public hospitals than in private hospitals [26]. The lack of psychiatric inpatient beds may result in longer waiting times in the emergency department [22], which may result in violent behavior. Caring is also related to violent behavior. Poor communication [19, 24] and lack of collaboration among healthcare providers during the patient-care process contribute to violent behavior [28].

Although several studies have shown that violence in emergency departments often involves psychiatric patients, the reported incidence rate of violent behavior among psychiatric emergency patients is limited. One study reported incidents of violent behavior among psychiatric emergency patients [3]. In addition, no incidents of violent behavior in emergency departments have been reported in Thailand. Moreover, a study on factors associated with violence in the emergency department focuses on patient-related factors. However, research on the multicomponent factors of service systems contributing to violent incidents in emergency departments is limited. Previous studies were often based on staff perceptions and were more subjective than objective. Herein, the incident rate of violent behavior among psychiatric emergency patients was explored, concerning the duration of the patient being boarded in the emergency department using an observational design. The impact of patient factors and psychiatric emergency service systems on violent behavior in the same model was also explained.Our results enhance the understanding of the mechanism by which patient factors and psychiatric emergency service systems affect violent incidents among psychiatric emergency patients in the general emergency department. Moreover, the findings can provide policy recommendations for improving emergency psychiatric service systems in general emergency departments, such as developing safety policies, enhancing emergency nursing skills, and promoting collaborative care between emergency and psychiatric services to enhance patient and staff safety.

## Methods

This study used a prospective observational design, focused on quantitative analysis, and was conducted in 16 emergency departments in central Thailand. Study participants were selected by stratified random sampling. The organizational variables were collected from emergency department personnel, and the data collection period was from March 2023 to October 2023.

### Setting

In central Thailand, four health service areas were identified, each consisting of hospitals of different sizes, which are classified based on their capacity. The regional hospital is a tertiary care facility that can accommodate complex patients who require specialized care and advanced technology. Large general hospitals can provide care for patients with more complex issues at highly specialized levels compared with small, general hospitals. The community hospital offers secondary care and accepts patients referred by primary care facilities. The service provision for psychiatric emergency patients at general emergency departments includes mental health triage, physical assessment, initial treatment to keep the patient calm, and care for psychiatric emergency patients. Patients experiencing psychiatric emergencies will be transported to the nearest general emergency department. The severity of their psychotic symptoms will be evaluated, and appropriate care will be provided to ensure that they are calm. Next, these psychiatric patients with sophisticated mental health conditions are referred to a high-level hospital in the health service area or a psychiatric hospital that can efficiently address their needs.

### Sample size

The sample size was calculated using the Cochran formula similar to Dawson et al.’s (2016) study to estimate the incidence of violence escalation in psychiatric emergency patients during care at the emergency department. Considering the required sample size of 156, a confidence level of 95% and an allowable error of 4.5% were used.

In adjusting errors using stratified sampling, Dattalo’s guidelines (2008) recommended establishing the design effect between 1 and 3. Thus, this study specified the design effect as 3. Consequently, the sample size was the sample group of psychiatric emergency patients (465 cases).

We used the rule of thumb based on the number of events per variable (EPV) to calculate the sample size for the Cox proportional hazard model. The EPV should be between 10 and 50. The required sample size was *n* = 100 + (30 × 2) = 160 cases, assuming an EPV of 30 for independent factors in the final model of 2. However, if stratified sampling with a design effect of 3 based on Dattalo guidelines was used, the sample size must be adjusted to 480 cases.

### Violence escalation and its measurement

Violence escalation is the level of violent behavior during a patient’s stay in the emergency department, which is higher than that at triage. The researcher and research assistant collected the data by observing the patients’ behavior during triage every 30 min and when the patient exhibited violent behavior. Each patient’s period from triage to violent behavior escalation—or 660 min (patients with no violent behavior escalation)—was documented for survival analysis. Violent behavior was measured using the overt aggressive scale (OAS), which was developed by Yudofsky et al. (1986) [29]. Herein, the researchers translated the OAS into Thai using back translation per Brislin’s guidelines. A bilingual psychiatric nursing professional translated the OAS into Thai and another expert translated it into English. Finally, content equivalence was conducted by a language expert. In addition, a score of 0 was assigned to indicate that the patient did not exhibit any behavior on the OAS. In Thai, the OAS assessed the severity of violent behavior as follows: (1) verbal aggression score of 0–4, (2) physical aggression against objects score range from 0–5, (3) physical aggression against self-score of 0–6, and (4) physical aggression against other people score of 0–6. The possible score ranges from 0 to 21.

Violence escalation was divided into two groups: (1) violence escalation indicates a violent behavior score higher than that assessed at triage of at least one part of the violent behavior. (0) Nonviolent escalation indicates a level of violent behavior that is less than or equal to the level of violent behavior assessed at triage.

### Patient variables and measurement

Patient variables were collected from patients over 18 years of age who presented to general hospital emergency departments with at least one of the following symptoms: violent behavior, verbally aggressive behavior, physically aggressive behavior, harm to self and others, agitation and restlessness with a psychiatric history, bizarre/disorganized behavior, delusions, hallucinations, paranoid, confusion, irritability, anxiety, and somatic symptoms with a history of mental illness, acute dystonia, parkinsonism, neuroleptic malignant syndrome, and medication-induced acute akathisia. Participants were excluded if they had at least one of the following symptoms: (1) need for resuscitation, (2) need for an incubation tube, (3) need for insertion of intercostal chest drainage, (4) Glasgow Coma score of ≤ 8, (5) oxygen saturation of < 90%, (6) life-threatening arrhythmia, (7) shock (systolic blood pressure of < 90 or mean arterial pressure of < 60 mmHg), (8) seizure, and (9) apnea.

Patient variables included age, physical and psychiatric comorbidities, transport methods, time of arrival, and emergency department disposition. Physical comorbidities indicate that psychiatric emergency patients are diagnosed with chronic illness or physical problems. Psychiatric comorbidities refer to the diagnosis of two or more psychiatric disorders by a psychiatrist or an emergency department physician. Methods of transport refers to how a patient is transported to the emergency department, which includes friends or relatives, the police, a foundation vehicle, and an emergency ambulance. Another patient variable is the time of arrival of patients at the emergency department. Emergency department disposition refers to the status or method by which an emergency psychiatric patient is discharged from an emergency department. Disposition is classified into three groups: discharge to home, hospitalization, and referral to another hospital. All patient variables were collected using the patients’ record form designed by the researchers (Supplementary 1).

### Delivery system variables and measurements of psychiatric emergency services

The form was developed to interview the head of the emergency department and three experts qualified for the same. The form was related to psychiatric emergency service system variables, including “the use of clinical practice guidelines,” which indicates that the emergency department used the guidelines in providing care for psychiatric emergency patients (Supplementary 2). This variable is divided into two groups: No (score = 0) and Yes (score = 1). “Availability of restraint devices” refers to the number and availability of straps or devices that limit patients’ freedom of movement by restraining the body. Herein, a ready-to-use restraint device indicates at least six straps or devices that the emergency department has for restraining the patient around the wrist or ankle. Such devices are complete and undamaged (score = 1). An unavailable restraint device indicated less than six pieces of straps or devices, or the straps were torn or damaged (score = 0). “Nurse productivity” is the proportion of nurses required and that can be provided for patients for nursing services. This variable was calculated with the following formula $$\frac{nursing care per hours per case visit}{the number of nurse \times 7} \times 100$$ [30]. “Policy clarification for emergency psychiatric patient care” clarifies the hospital’s policy in improving emergency psychiatric care. We used the assessment form provided by the psychiatric emergency service system. The hospital policy was adapted from the assessment form of the emergency medical care systems of different hospitals [31], along with the care policy for psychiatric patients in crisis from the Department of Mental Health. The assessment form consists of five questions with six choices. The scores range from 0 to 25, with high scores indicating that the policy is clear and can be implemented. “A dedicated room for emergency psychiatric care” is an emergency department with a separate room or area for patients who need emergency psychiatric care. This study divided the service areas into emergency departments without (score = 0) and with (score = 1) an emergency department with a separate area for emergency psychiatric patient care. “Emergency room overcrowding” indicates that the care needs of patients in the emergency department exceed its capacity to provide services. This lack of capacity was assessed using the emergency department work index (EDWIN) developed by Bernstein et al. (2003) [32]. The size of the hospital is classified per its capacity to provide services. Based on the criteria for the level of health services of hospitals under the Office of the Permanent Secretary, Ministry of Public Health Thailand, hospitals are classified as follows: regional, sizable general, small general, and community hospitals. “The number of psychiatric inpatient beds” refers to the number of psychiatric hospital beds or beds reserved for psychiatric emergency patients in each hospital. “The proportion of psychiatrists per population” is the number of psychiatrists or physicians approved for preventive medicine certificates (Community Psychiatry) who consult psychiatric emergency patients from hospital emergency departments per 100,000 population in responsibility. “The model of emergency psychiatric patient care” is a method for consulting a psychiatric specialist to assess a patient. Consultation is classified into four types—no consultation, telephone consultation, psychiatric specialist visiting a patient in the emergency department, and telephone consultation and psychiatric specialist visiting a patient in the emergency department.

In addition, organizational variables related to the emergency nurse included nurse competency and use of de-escalation. Nurse competency is the perception of an emergency nurse to care for psychiatric emergency patients. This study assessed nurse competency using the Thai version of behavioral healthcare competency (BHCC in Thai). This five-point Likert scale questionnaire consists of 23 questions. A higher score indicates higher perceived competency in providing care to psychiatric emergency patients. The researcher translated the BHCC scale developed by Rutledge et al. (2012) [33] into Thai with back translation per Bristlin’s guideline [34]. The Thai version of BHCC was qualified for reliability and yielded an alpha Cronbach coefficient of 0.947. “The use of the de-escalation technique” refers to the communication behavior of emergency nurses to minimize the severity of patients with aggressive behavior. Communications were assessed using the English-modified De-escalating Aggressive Behavior Scale (EMDABS in Thai). The researcher translated the EMDABS scale developed by Mavandadi et al. (2016) [35] into Thai with back translation in accordance with Bristlin’s guidelines [34]. The Thai version of EMDABS was assessed for reliability, and the alpha Cronbach coefficient was 0.835. The scale consists of seven questions, with five choices (1 = strongly disagree and 5 = strongly agree). The scores range from 7 to 35. A high score indicates good behavior in reducing violence.

### Data collection

First, data on psychiatric emergency service systems were collected by conducting structured interviews with the head of the emergency department using the psychiatric assessment form of the emergency service system. This form consists of a standard set of questions explained in the delivery system variables and measurement section. A total of 16 emergency department heads were willing to participate in this study (100%), apart from collecting data from registered nurses who work in emergency departments—including nurse competency and the use of the de-escalation technique. The registered nurses were given consent forms to collect data on nursing competency. Nurses answered the self-report questionnaire BHCC to assess competency perception in providing care for psychiatric emergency patients. A total of 236 registered nurses with > 2 years of experience in the emergency department were willing to participate. Of these, 41.5%, 33.5%, 18.6%, and 6.4% were from regional, large general, small general, and community hospitals, respectively. Second, the researcher solicited nurses from the emergency departments as research assistants. The research assistants were prepared by explaining the research details and the data collection process using the OAS to observe violent behavior and patient record form to collect patient information, as well as the number of emergency patients and nurses and physicians who worked the same shift as the psychiatric patient waiting in the emergency department. The researcher trained the research assistants until they were reliable. Third, the researcher and research assistant asked the emergency nurse who cares for emergency patients in the waiting room to attempt EMDABS in Thai after caring for psychiatric emergency patients. The response rate is 94.5%, which accounts for 479 psychiatric patients with a nurse willing to participate.

### Ethics

This study was reviewed and approved by the Institutional Review Board Faculty of Nursing Mahidol University (COA No. IRB-NS2023/754.1602) and the committees of selected hospitals under the Ministry of Public Health. Written informed consent was obtained from the emergency department head and nurse. Legal guardians provided consent for psychiatric emergency patients with psychotic symptoms or behavioral problems. Psychiatric emergency patients without legal guardians were not included in the study. All procedures were performed per the ethical guidelines and regulations.

### Statistical analysis

Descriptive statistics described the characteristics of the participants and the delivery systems of psychiatric emergency services. Either Pearson chi-square test or Fisher’s exact test was performed to compare the proportions of participants with and without violence escalation. The Mann–Whitney U test was used to compare the median between the two groups.

Herein, the Kaplan–Meier method estimated the curve and rate of escalation-free violence. The log-rank test compared the curve of escalation-free violence between different levels of violent behavior across groups. Univariable and multivariable Cox proportional hazard models analyzed the patient and psychiatric emergency service delivery systems associated with the time of escalation-free violence. The unadjusted hazard ratio (HR) and adjusted hazard ratio (aHR) along with their corresponding 95% confidence interval (95% CI), determined the strength and direction of the association. Data were recorded and analyzed using IBM SPSS Statistics for Windows, Version 27.0, released in 2020 by IBM Corp. *P*-values < 0.05 were considered statistically significant.

## Results

A total of 507 psychiatric emergency patients were included in this study and 33.3%, 34.5%, 11.4%, and 20.7% (*n* = 169, 175, 58, and 105) were from four Reginal, four large general, two small general, and six community hospitals, respectively. In addition, 64.4% of the participants were sent to the emergency department by a friend or relative, 20.8% by an ambulance, and 14.8% by the police. Self-harm was the predominant reason for emergency department visits for 24.1% of the participants, followed by verbal aggression (15%) and violent behavior (13.2%). Based on the Thai emergency severity index, most participants (65.6%) classified the severity of their symptoms as level 2.

### Incidence rate of violence escalation

A total of 507 psychiatric emergency patients were included in this study, and the violence score from the triage was increased for 37 of them. The overall cumulative incidence of violence escalation was 7.3%. The small general hospital had the highest cumulative incidence (15.52%), followed by the large general (7.4%), regional (7.1%), and community (2.8%) hospitals. In addition, 37 patients experienced violence escalation. The patients’ time at risk of violence escalation was 70,496 min. The incidence rate of violence escalation is calculated as follows:

$$\begin{array}{c}=\frac{number \,of \,new \,cases \,of \,violence \,escalation}{Patient-time \,at \,risk \,of \,violence \,escalation},\\ = \frac{37 persons}{\text{70,496} \,minute} = 0.000524852\text{ persons}/\text{person}-\text{min},\\ = 0.03\text{ people}/\text{person}-\text{h or }3\text{ people}/100\text{ person}-\text{h}\end{array}$$

Based on the study results, the rate of violent behavior escalation was 3 people per 100 person-h. Escalating violence indicates that every hour, 100 psychiatric emergency patients visit the emergency department, and 3 of these patients exhibit increasing levels of violent behavior.

### Characteristics of psychiatric emergency patients with and without violence escalation

More than 50% of the participants in the violence escalation group were male. The median age was 31.5 years (19–69 years). Most of the participants, with and without escalating violence, did not have any psychiatric comorbidities. The participants in the violence escalation group had a higher percentage of psychiatric illnesses (66.7%) than those in the nonviolence group (54.1%). Similarly, the group that escalated violence had a higher percentage of substance use (47.2% vs. 40.3%) than the group that did not escalate violence. Concerning the severity of violent behavior, the violence escalation group had a median violent behavior score of 1, which was higher than that of the nonviolence escalation group. When comparing participant characteristics between the violence and nonviolence escalation groups, the statistics revealed that only the levels of violent behavior significantly differed (*p* = 0.018, Table 1).

**Table 1:** Comparing the characteristics of participants between violence escalation and non–violence escalation

Characteristics of the participants	Violence escalation n (%)		*P*—value
	**Non – violence** ***n* = 470 (92.7%)**	**Violence** ***n* = 37 (7.3%)**	
**Sex **^**a**^			0.428
*Male*	260 (55.4%)	23 (62.2%)	
*Female*	209 (44.6%)	14 (37.8%)	
**Age **^**a**^	Median 34.00 Range (18 – 93)	Median 31.50 Range (19 – 69)	0.587
**Comorbidity **^**a**^			0.873
*No*	343 (73.4%)	26 (72.2%)	
*Yes*	124 (26.6%)	10 (27.8%)	
**Psychiatric comorbidity **^**a**^			1.000
*No*	426 (91.8%)	33 (94.3%)	
*Yes*	38 (8.2%)	2 (5.7%)	
**History of psychiatric illness**^**a**^			0.144
*No*	214 (45.9%)	12 (33.3%)	
*Yes*	252 (54.1%)	24 (66.7%)	
**Substance use **^**a**^			0.416
*No*	277 (59.7%)	19 (52.8%)	
*Yes*	187 (40.3%)	17 (47.2%)	
**Time arrival **^**a**^			0.529
*Morning shift*	230 (48.9%)	21 (56.8%)	
*Afternoon shift*	197 (41.9%)	12 (32.4%)	
*Late night shift*	43 (9.1%)	4 (10.8%)	
**Severity score (ESI level) **^**a**^			0.899
*ESI 2*	308 (65.7%)	24 (64.9%)	
*ESI3*	142 (30.3%)	12 (32.4%)	
*ESI4*	19 (4.1%)	1 (2.7%)	
**Level of violent behavior at triage**^**b**^	Median 0 Range 0—18	Median 1 Range 0 – 10	0.018*

^a^comparison using the Chi-square test

^b^comparison using the Mann – Whitney U test

### Characteristics of the psychiatric emergency service delivery system

The characteristics and factors of the psychiatric emergency service delivery system in the violence escalation group were considered. The median nurse competency score in the violence escalation group was lower than that in the nonviolence escalation group (median 3.24 vs. 3.28). The proportion of psychiatrists per 100,000 people in the violence escalation group was 0.65, which was the same as that in the nonviolence escalation group. The clinical practice guidelines were used by 27% of the violence escalation group. By contrast, nearly half (47.9%) of the nonviolence escalation group used clinical practice guidelines. The availability of restraint devices in the violence escalation group was 51.4%, which is approximately equal to that in the nonviolence group (51.5%). The violence escalation group had a lower percentage of dedicated areas for psychiatric emergency patients than the nonviolence escalation group (2.7% vs. 17.9%). The median number of psychiatric inpatient beds was 8, which was equal to that in the violence and nonviolence escalation groups. The median score for the clarification psychiatric emergency care policy in the violence escalation group was equal to the nonviolence escalation group (median 10, range 0–16 vs. median 10, range 3–16). The median score for emergency overcrowding in the violence escalation group was lower than that in the nonviolence escalation group (EDWIN score 2.97 vs. 3.13). The median score for nurse productivity in the violence escalation group was lower than that in the nonviolence escalation group (88.10 vs. 114.8).

In the violence escalation group, most psychiatric emergency care models (51.4%) involved phone consultations or visits by specialists to evaluate the patient in the emergency department. Conversely, no psychiatric consultation was reported in the nonviolence escalation group (45.7%). The median de-escalation technique score of the nonviolence escalation group was 32 (range 11–35), which was the same as that of the nonviolence escalation group, with a median score of 32 (21–35). Most patients in the violence escalation (62.2%) and nonviolence (54.5%) groups were discharged from the emergency room to the inpatient wards. Furthermore, the violence escalation group stayed more in the emergency room than the nonviolence escalation group (147 vs. 102 min).

### Patient and psychiatric emergency service delivery factors associated with violence escalation

**Fig. 1:**
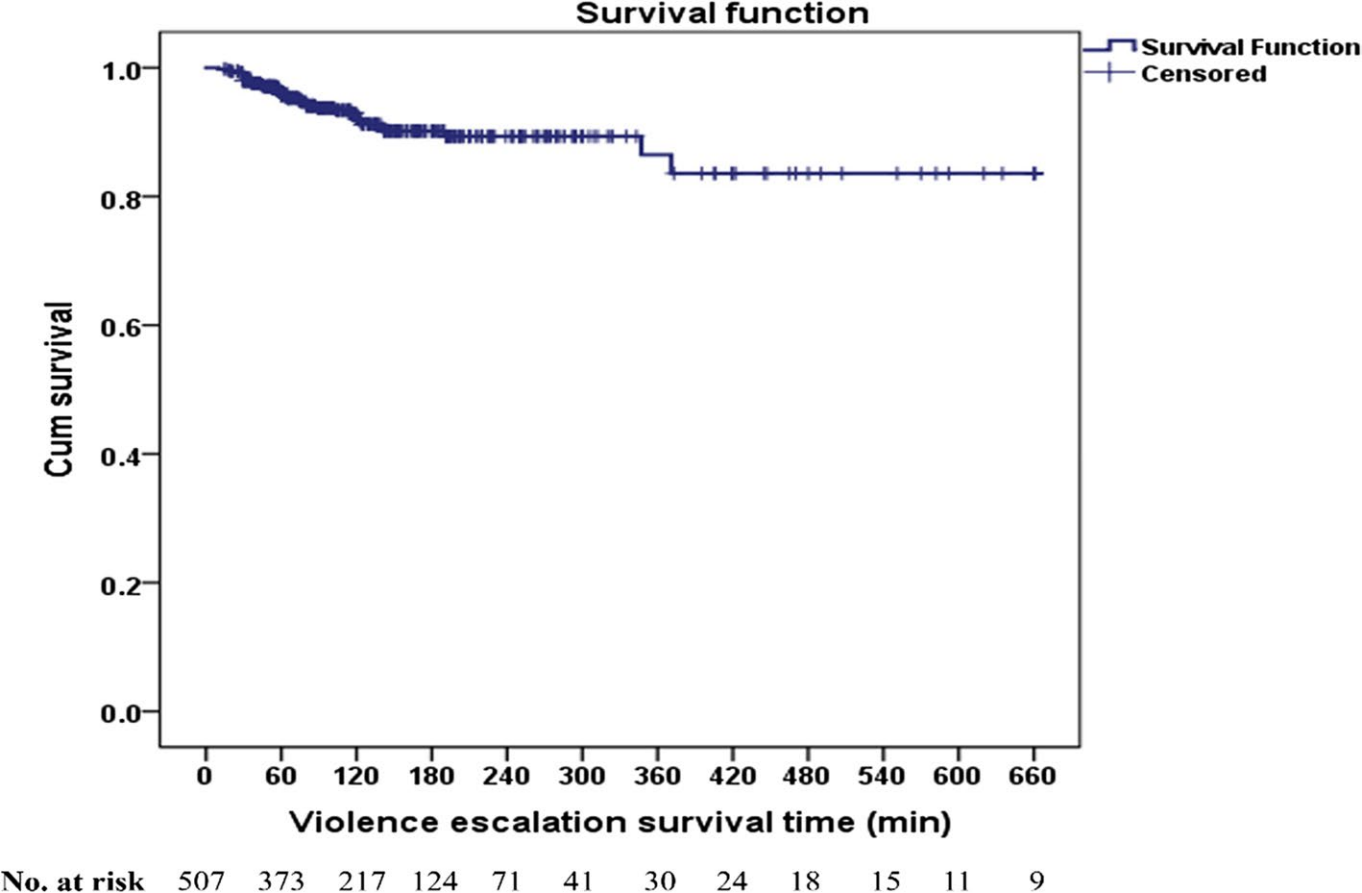
Violence escalation-free time among 507 psychiatric emergency patients

The Kaplan–Meier method was used to analyze the survival time of violence escalation. The violence escalation-free rate was 83.6%. However, > 50% of the patients did not experience violence escalation, indicating the absence of median free time for violence escalation. Psychiatric emergency patients under observation at the beginning and at 120, 240, 360, 480, and 660 min were 507 (100%), 217 (42.8%), 71 (14.0%), 30 (5.9%), 18 (3.5%), and 9 (1.8%), respectively (Fig. 1).

Univariable analysis of the association between patient and psychiatric emergency service delivery factors and violence escalation-free time using the Cox proportional hazard model was performed to investigate patient factors associated with violence escalation-free time. The level of violent behavior at triage was remarkably associated with the survival time of violence escalation (*p* = 0.034, Table 2).

**Table 2:** The association of survival time of violence escalation and patient factors by univariable analysis: Cox proportional model

Patient factors	n	Unadjusted HR	95%CI	*p*-value
Age		0.989	0.965—1.014	0.393
**comorbidity**				
*No*	369			
*Yes*	134	0.923	0.444—1.917	0.829
**psychiatric comorbidity**				
*No*	459			
*Yes*	40	0.621	0.149—2.589	0.513
**Level of violent behavior**				
*OAS score* = *0*	341			
*OAS score* ≥ *1*	165	2.009	1.053—3.834	0.034*
Time of arrival				
*morning shift*	251			0.504
*afternoon shift*	209	0.779	0.383—1.586	0.492
*late night shift*	47	1.510	0.515—4.428	0.453
**Type of disposition**				
*Discharge to home*	158			0.445
*Admit*	278	1.732	0.742—4.045	0.204
*Refer*	71	1.479	0.514—4.259	0.468

Moreover, the psychiatric emergency service delivery factors associated with the survival time of violence escalation included nurse competencies (*p* = 0.014), the use of clinical practice guidelines (*p* = 0.037), and hospital size (*p* = 0.026, Table 3).

**Table 3:** The association of survival time of violence escalation and psychiatric emergency service delivery factors by univariable analysis: Cox proportional model

Psychiatric emergency service delivery factors	n	Unadjusted HR	95%CI	*p*-value
**Nurse competency**		0.152	0.034—0.685	0.014*
**The proportion of psychiatrist**		0.912	0.559—1.489	0.712
**Using clinical practice guideline**				
*Not Used*	272	reference		
*Used*	235	0.461	0.223—0.954	0.037*
**Availability of restraint devices**				
*Unavailability*	261	reference		
*Availability*	246	0.998	0.523—1.905	0.996
**Nurse Productivity score**		0.995	0.989—1.002	0.184
**Policy clarification score**		1.014	0.953—1.079	0.660
**Having an area for emergency psychiatric care**				
*No*	422	reference		
*Yes (Corner)*	85	0.154	0.021—1.125	0.065
**Emergency room overcrowding**				
*Can manage*	58	reference		0.696
*Busy*	60	0.549	0.137—2.195	0.396
*Overcrowding*	384	0.841	0.347—2.035	0.701
**Hospital size**				
*Regional hospital*	169	reference		0.026*
*Large general hospital*	175	1.467	0.662—3.250	0.345
*Small general hospital*	58	2.386	1.005—5.666	0.049*
*Community Hospital*	105	0.361	0.102—1.281	0.115
**Number of psychiatric inpatient bed**		1.035	0.997—1.074	0.068
**Mode of arrival**				
*Friend/relative*	326	reference		0.748
*Police*	75	0.664	0.231—1.907	0.446
*Ambulance*	105	0.964	0.434—2.141	0.928
**Model of psychiatric emergency care**				
*No psychiatric consultation*	226	reference		0.235
*Telephone consultation*	34	0.443	0.164—1.202	0.110
*ED visit*	202	0.213	0.026—1.769	0.152
*Telephone and ED visit*	45	0.738	0.294—1.847	0.516
**Using de-escalation technique**		1.007	0.937—1.082	0.851

Based on the univariable Cox regression analysis, four factors were associated with violence escalation-free time, including the level of violent behavior at triage, nurse competency, use of clinical practice guidelines, and hospital size. These four factors were included in the multivariable Cox regression analysis using Forward LR. The results indicated that only two independent factors were significantly associated with violence escalation-free time—level of violent behavior at triage and nurse competency—while controlling other covariate factors. Patients with a violent behavior score of ≥ 1 at triage had a two times higher risk of violence escalation than those with a score of 0 at triage (95% CI 1.051–3.823, *p* = 0.035). Furthermore, a one - unit increase in nurse competency is related with an 85.3% reduction in the risk of violent escalation (95% CI 0.032–0.680, *p* = 0.014; Table 4).

**Table 4:** Univariable and multivariable Cox regression analyses of factors and the survival time of violence escalation

Independents factors	Unadjusted HR (95%CI)	*p*-value	Adjusted HR (95%CI)	*p*-value
**Level of violent behavior**				
*OAS score* = *0*	reference		reference	
*OAS score* ≥ *1*	2.009 (1.053—3.834)	0.034	2.004 (1.051—3.823)	0.035
**Nurse competency**	0.152 (0.034- 0.685)	0.014	0.147 (0.032—0.680)	0.014

## Discussions

Herein, the cumulative incidence of violence escalation among psychiatric emergency patients who visited the general emergency department in central Thailand was 7.3%—lower than that of Dawson et al.’s (2018) study on psychiatric patients who exhibited aggressive or violent behavior during their stay in the general emergency department (9%). This difference is potentially because the present sample spent less time in the emergency department than the sample in Dawson’s study. We discovered that the median length of stay in the emergency department was 107 min (range 15–2,621 min). Moreover, the median length of stay for patients with and without violence escalation was 147 and 102 min (range: 50–761 and 15–2,621 min). Dawson’s study of psychiatric emergency patients boarded for 72 h (4,320 min), with a median length of stay of 655.5 and 376 min for violent and nonviolent patients [3].

The first study investigated the incidence rate of violence escalation. The incidence rate of violence escalation was 3 per 100 psychiatric emergency patient visit hours, which was lower than that in a previous study. Kleissl-Muir et al. (2018) conducted a scoping review of workplace violence in the emergency department. Their findings indicated that the incidence of violence in this setting was 2.8–10.3 incidents per 1,000 visits to the emergency department [19].

The level of violent behavior at triage predicted violence escalation. If the psychiatric emergency patient had a violent score of ≥ 1 at triage, this patient exhibited behavior of making a loud voice or screaming with anger. A twofold increased risk of violence escalation was observed in patients who did not exhibit violent behavior at triage. Screaming is a sign of escalating tension; if the patient is not de-escalated through verbal or behavioral interventions, such as communication to calm the patient or keeping the patient in a quiet place, the behavior may escalate to aggressiveness and violence [36]. Moreover, the psychiatric emergency patient may have a perpetuating factor of violence, such as psychotic symptoms, which are dynamic and can change over time [36]. Psychotic symptoms can make a patient feel uncontrolled and unsafe, which is linked to violent behavior [37]. Herein, we found that patients who exhibited verbal aggression or threatening behavior during triage were at risk of violence in the emergency department, which is consistent with prior research. Partridge and Affleck (2018) discovered that 34.3% of violent patients had been verbally threatened at the triage area, and patients with a violent score of ≥ 1—when assessed using the Broset violence checklist—were 11.6 times more likely to commit violence [38]. Furthermore, Kim et al. (2022) reported that exhibiting aggressive behavior or making threats at triage significantly increases the probability of violence by 24 times (95% CI 7–80) [2].

Nurse competency predicts violence escalation. A one - score increase in nursing competency reduces the likelihood of violent escalation by 85.3%. Competence refers to the capacity to effectively provide service to patients by combining knowledge, skills, attitudes, and decision-making [39]. Nurses with these skills can reduce the incidence of violent behavior. This study is the first to demonstrate an association between violent behavior and emergency nursing competence in caring for patients with behavioral problems. Nevertheless, Pich et al. (2017) found that 60% of nurses considered the lack of ability to deal with psychiatric patients as a contributing factor to violence in the emergency department. In addition, the lack of psychiatric knowledge and training for emergency nurses contributed to the increase in violent behavior [19]. Moreover, a study examining the competence of emergency nurses using the BHCC survey revealed that nurses who encountered violence in their workplace had a heightened perception of their performance than those who had never experienced violence [40]. The authors of this study concluded that enhancing nurses’ performance and operational skills is essential in improving therapeutic care for patients with mental illness who come to the emergency department and in promoting the safety of patients and staff [40].

Psychiatric emergency service delivery factors include the use of clinical practice guidelines, availability of restraint devices, nurse productivity, policy clarification, having a dedicated area for psychiatric emergency patients, emergency room overcrowding, hospital level, number of psychiatric inpatient beds, proportion of psychiatrists per population, model of psychiatric emergency care, and use of the de-escalation technique, which was not associated with violence escalation.

The use of clinical practice guidelines was not associated with violence escalation, which is inconsistent with the findings of Sharifi et al. in 2020 [41], who found that implementing preventive protocols in the emergency department remarkably reduced the incidence of violence. This discrepancy could be because most of the clinical practice guidelines in the setting of this study focused on clinical workflow without guiding the prevention of violent behavior escalation. This clinical emphasis is contrary to the preventive protocol used in the Sharifi study, which included a clear practice guideline for communicating with patients and adding the protocol to the medical record, further leading to personal adherence. The availability of restraint devices was not associated with violence escalation. This finding is contrary to the results of Pich et al. (2017), who found that restraint predicted the incidence of violence in emergency departments [24]. The correlation between restraint and violence may depend more on the process of restraining patients than on the equipment used. A previous study showed that patients perceived restraint as a form of punishment [42] and did not receive any communication from the provider regarding the reason for their restraint [43]. Therefore, inappropriate use of restraints may cause violent behavior. No dedicated area for emergency psychiatric care was associated with violence escalation. This nonassociation may be due to the fact that > 80% of the study setting had no dedicated area—only 12.5% had a designated area, which was merely a corner within the main emergency room. This arrangement exposed patients to the general emergency department environment, which could explain the lack of association between a dedicated area for emergency psychiatric care and violence escalation.

Reportedly, overcrowding in emergency departments can lead to violent incidents [23, 25]. However, this study found no such association because emergency room overcrowding was measured by the number of patients who visited the emergency department in the same shift as psychiatric patients, which did not reflect emergency overcrowding when the patient was in the emergency department. Moreover, this study revealed that nursing productivity was not associated with violence escalation, which contradicts the findings of a previous study [19, 24, 44]. This difference can be explained as follows: psychiatric emergency patients in this study receive care from nurses and assistant nurses or nurse aids. Nursing productivity calculated using only the number of emergency nurses cannot accurately predict violent behavior. This finding is supported by Staggs (2015), who found that the number of hours per patient’s day for nonregistered nurses is significantly associated with the rate of assault in inpatient psychiatric units [45]. Clarification of the policy was not associated with violence escalation potentially because our study focuses on developing the structure of psychiatric emergency service systems rather than preventing violence. This emphasis differs from previous studies reporting that having a policy focusing on security and preventing violence is crucial to avoid violent incidents in the emergency department [19, 46].

The number of psychiatric inpatient beds was not associated with violence escalation, probably because the correlation between the number of inpatient beds and violence escalation depended on waiting time in the emergency department. Similarly, previous research has shown that the lack of inpatient beds can lead to longer waiting times for psychiatric emergency department patients [22], which can increase the likelihood of violent incidents occurring [47].

Based on our analysis using univariable Cox regression, a link between violence escalation and hospital size was initially discovered. However, when multivariable Cox regression analysis was conducted, hospital size was an insignificant factor influencing violence escalation, indicating that other independent factors may play a more important role in violence escalation than hospital size. This observation is consistent with the SEIP 2.0 framework, which proposes that the different components of a work system collectively affect the work process and that only specific components may interact with the work process and its outcomes, depending on their influence in the work system [21].

Furthermore, no significant correlation was found between the proportion of psychiatrists and violence escalation. However, this association may depend on the waiting time for psychiatric assessment. Stone et al. (2012) showed that the lack of resources for psychiatric assessment can lead to patients being held in the emergency room for extended periods [22], and longer waiting times have been linked to violent behavior in psychiatric patients [3]. Herein, the median time between patient arrival and psychiatric assessment was 40.5 min, which is relatively shorter than that in Weiss et al.’s (2012) study [48]. The emergency psychiatric patient-care model was not associated with violent behavior. These findings contradict the systematic review conducted by Evans et al. (2019), who discovered that several models of psychiatric emergency care reduced the use of seclusion and restraint in response to the risk of violent behavior [49].

The use of the de-escalation technique was not associated with violence escalation; this finding was inconsistent with the results of a randomized controlled trial, which found that de-escalation remarkably reduced violence incidence in acute psychiatric units [50]. Herein, de-escalation was not associated with violence escalation, probably because this technique is performed before a patient becomes violent. However, violent patients commonly exhibited aggressive behavior at triage during our study. Nurses had to manage their behavior through restraint or psychiatric medication to ensure the safety of patients and providers.

### Strengths and limitations

No study has investigated violence escalation in psychiatric emergency patients by examining the patient and service delivery systems in the same model. Therefore, this study is the first to explore these two factors simultaneously, and the results showed that patient factors and service delivery systems were significantly associated with violence escalation in psychiatric emergency patients.

A limitation of this study was the reliability of the testing process of violent behavior observation tools. The researcher did not test the interrater reliability of the OAS because of the uncertain timing of psychiatric patients’ access to the emergency department and the multisetting nature of the study. However, the researchers clarified the use of the OAS for research assistants to observe violent behavior. Moreover, the research assistant can contact the researchers at any time during data collection if there are any uncertainties.

Second, providing a practical definition of violence escalation was challenging. Herein, violence escalation is defined as the high score of patients’ violent behavior during their stay in the emergency department compared with that at triage. This definition excludes patients with a high violent behavior score at triage. For example, the patient made verbal threats and hit the bed at triage; consequently, the patient was restrained in managing their behavior. Subsequently, the patient waited in the emergency department and expressed his frustration by shouting. Behavioral scores assessed during the patient’s stay in the emergency department were lower than those during triage, which were not indicators of violence escalation. These limitations may impact the incidence of violence.

Third, collecting data on real-time psychiatric emergency service delivery factors, such as emergency overcrowding and nurse productivity, is difficult. Herein, data were collected by recording the number of patients and providers present during the same shift in the psychiatric emergency department. This data collection method may affect the investigation of the association between the aforementioned factors and violence escalation. However, many studies have indicated that overcrowding and nurse productivity have considerable effects on violence in emergency departments, indicating that these factors should be investigated rigorously.

Finally, although this study was conducted in Thailand, the research findings can be generalized internationally because violence in emergency departments is widespread, and several studies have found similarities across different locations [51].

## Conclusion

The incidence of violence escalation among psychiatric emergency patients who visited the general emergency department in the central part of Thailand was 7.3%, and the incidence rate was 3 per 100 psychiatric emergency patients visits per hour. The level of violent behavior at triage with an OAS score of ≥ 1 and nurse competency to care for psychiatric emergency patients were considered as crucial factors that reduce the risk of violence escalation. Accurate triage of violent behavior in emergency departments can prevent violence escalation and monitor violent behavior by providing an early intervention. Furthermore, knowledge, attitude, and practical skills are necessary to care for psychiatric emergency patients in the emergency department.

### Supplementary Information

Supplementary Material 1.

Supplementary Material 2.

## Data Availability

No datasets were generated or analysed during the current study.

## Abbreviations

aHR: Adjusted hazard ratio
EPV: Events per variable
OAS: Overt Aggressive Scale
EMDABS: English-modified De-escalating Aggressive Behavior Scale
BHCC: Behavior Health Competency Care
EDWIN: Emergency Department Work Index
