# Supplementary material for: Incident of violence escalation of patients with psychiatric emergencies boarding in the emergency department in the central region of Thailand and its association: a prospective observational study
Source: BMC Health Serv Res. 2024 Jun 27;24:768. doi: 10.1186/s12913-024-11228-0 (PMC11210055; doi:10.1186/s12913-024-11228-0)
Supplement: Supplementary file 1 — Supplementary Material 1. [file 12913_2024_11228_MOESM1_ESM.docx]

**Record forms for emergency psychiatric patients in the emergency departments.**

**Explanation:** This form is designed to collect data on psychiatric patients who visit the general emergency department in case of an emergency. The researcher or research assistant checks ✓ in the box whether the information provided matches the patient's details or fills in the answer entirely in the designated space.

**Part 1:** **The general information of the emergency psychiatric patient**

1.1 Code……………..

1.2 Sex ❑ Female ❑ Male

1.3 Age ...........years

1.4 Chief complaint

❑ Violence behavior ❑ Verbal aggression ❑ Physical aggression ❑ Intention self-harm

❑ Harm to others ❑ Bizarre/disorganized behavior.

❑ Having a psychiatric illness with agitation or restless

❑ Delusion, Hallucination, Paranoid ❑ Confusion

❑ Irritability ❑ Anxiety ❑ Somatization

❑ Other……………………………………………………………………………

1.5 History of psychiatric illness ❑ No ❑ Yes, please specify...................................

1.6 Psychiatric comorbidity ❑ No ❑ Yes, please specify....................................

1.7 Physical comorbidity ❑ No ❑ Yes, please specify....................................

1.8 History of substance used.

❑ No

❑ Yes, please specify the type of substance.

1)……………………. Duration…………… (month/year)

2)…………………. ... Duration…………… (month/year)

1.9 Currently used substance.

❑ No ❑ Yes, please specify the type of substance.

1)……………………. Duration…………… (month/year)

2)…………………. ... Duration…………… (month/year)

1.10 Diagnosis.................................................................................................

**Part 2: the data on emergency services used by psychiatric patients.**

2.1 The number of times a patient visited the emergency department in the last 12 months ………times

2.2 Method of transporting patients to the emergency department.

❑ Friend/Relative ❑ Police

❑ Foundation ❑ Ambulance

❑ Patient own ❑ other…….

2.3 Date and time of patient arrival: date……………………….time………………………

2.4 Date and time of triage

2.4.1 Time of triage ***..................***

2.4.2 Emergency Severity Score

❑ ESI level 1 ❑ ESI level 2 ❑ ESI level 3 ❑ ESI level 4 ❑ ESI level 5

- 1. **Psychiatric emergency care.**
     1. Time of assessment……………………………………….…………….

1. Personnel ❑ Physician ❑ Emergency nurse
   - 1. Lab test

❑ Yes

- Time of investigation by lab test…………….
- Type of Lab test 🔾 CBC 🔾 U/A 🔾 BUN 🔾 Cr 🔾 Blood sugar

🔾 Electrolyte 🔾 LFT 🔾 Urine for substance

🔾 Chest X-ray 🔾 Other……………………

❑ No

- - 1. Receiving treatment in the emergency department.

1. Physical treatment

🔾 Dressing or suture 🔾 Splint 🔾 NG lavage

🔾 Antidotes for poisoning 🔾 Oxygen therapy

🔾 Other………………………………..

1. Psychiatric treatment

🔾 Haloperidol 5 mg, please specify

🞎 Oral, please specify time……. /………./…….

🞎 Intramuscular injection, please specify time…. /….../…….

🔾 Diazepam 10 mg

🞎 Oral, please specify time……. /………./…….

🞎 Intravenous injection, please specify time……. /………./…….

🔾 Other, please specify time……. /………./…….

- 1. **Psychiatric assessment**
     1. Receiving psychiatric assessment

❑ Yes, please specify time………….

❑ No

- - 1. Psychiatric specialist who assesses the patient

🔾 Psychiatrist 🔾 Psychiatric nurse

🔾 Emergency nurse who trained in a nursing specialty in psychiatric

nursing

🔾 other…………………………………………………………

- - 1. Method of assessing the patient.

🔾 Telephone 🔾 Telemedicine.

🔾 Visit the patient in the emergency department.

- 1. **Discharging a patient**
     1. Time of disposition …………………….
     2. Time of patient leave from emergency department………….
     3. The personnel who decide to discharge the patient.

🔾 Physician 🔾 Psychiatrist

🔾 Emergency nurse 🔾 Psychiatric nurse

- - 1. Type of disposition

🔾 Discharge to home 🔾 Admit to inpatient ward

🔾 Refer to a psychiatric hospital 🔾 Refer to a higher-level hospital.

🔾 Other………………………………………………………………

**Part 3:** Information about emergency department services during the psychiatric patient's stay in the emergency department.

3.1 Date…………………………………….

❑ Morning shift (08:00 AM – 6:00 PM)

❑ Afternoon shift (06:01 PM – 12:00 AM)

❑ Late night shift (12.01 AM – 07:59 AM)

- 1. The number of emergency patients who stay in the emergency department simultaneously as emergency psychiatric patients.
- ESI level 1........................patients
- ESI level 2........................patients
- ESI level 3........................ patients
- ESI level 4........................ patients
- ESI level 5........................ patients
  1. the number of attending physicians on duty……………………………………….
  2. The number of patients who wait for admission………………………………….

(During psychiatric patient stay in the emergency department)

- 1. The number of beds for treatment in the emergency department………………….

(During psychiatric patient stay in the emergency department)
