# Supplementary material for: Incident of violence escalation of patients with psychiatric emergencies boarding in the emergency department in the central region of Thailand and its association: a prospective observational study
Source: BMC Health Serv Res. 2024 Jun 27;24:768. doi: 10.1186/s12913-024-11228-0 (PMC11210055; doi:10.1186/s12913-024-11228-0)
Supplement: Supplementary file 2 — Supplementary Material 2. [file 12913_2024_11228_MOESM2_ESM.docx]

**psychiatric emergency service system assessment form**

**Part 1: The general characteristics of hospital and emergency departments.**

**Explanation:** The form was developed to interview the head of the emergency department regarding the delivery of psychiatric emergency services. Please check ✓ into ❑ or fill out the data.

1.1 What is the level of the hospital?

❑ Regional hospital

❑ Large general hospital

❑ Small general hospital

❑ Community hospital

- 1. How many inpatient beds? ………………………. beds

(Number of inpatient beds for male……. beds, number of inpatient beds for female …. beds)

- 1. Does the hospital have a psychiatric inpatient ward?

❑ Yes,

🔾 Having a psychiatric ward, please specify the number of psychiatric inpatient beds.………beds (Female…. beds, Male…. beds)

🔾 Having inpatient psychiatric beds within a medical ward, please specify the number of inpatient beds………. beds

❑ No

1.4 The number of emergency patients who visited emergency departments during the study period.

The number of patients in the ESI level 1 =………

The number of patients in the ESI level 2 =………..

The number of patients in the ESI level 3 =………..

The number of patients in the ESI level 4 =………..

The number of patients in the ESI level 5 =………..

- 1. The number of emergency nurses =………

**Part 2: The psychiatric emergency services delivery systems.**

**Explanation:** The form was developed to interview the head of the emergency department regarding the delivery of psychiatric emergency services. Please check ✓ into ❑ or fill out the data.

2.1 Does the emergency department use clinical practice guidelines for caring for psychiatric emergencies?

❑ No ❑ Yes, please provide the name of the guidelines……………………

2.2 The devices and medication for restraining psychiatric emergency patients.

- Please provide information about the number of restraint clothes.……………….
- Please provide information about the type and number of any additional devices used for restraint.……………………………………………………………………………
- Are medicines used to restrict patient behavior stored in the emergency department?

❑ Yes, please specify

🔾 Diazepam …………. Amp 🔾 Haloperidol …………Amp

🔾 Other………………..

2.3 "Is there a designated room for psychiatric emergency patients in the emergency department?

❑ No

❑ Yes, please specify the characteristics of the designated room.

🔾 Dedicated room, Size…………………. Square meters

🔾 Dedicated area, Size…………………. Square meters

2.4 What is the proportion of psychiatrists per population?

2.4.1 the proportion of psychiatrists per population..................per population

2.4.2 Having the Psychiatrist for consultation.

❑ No

❑ Yes, please specify.

🔾 The number of psychiatrists in the hospital…………….

🔾 The number of psychiatrists from another hospital within the health area

- The name of the hospital………………………
- The number of psychiatrists……………………

2.5 What is the model of psychiatric emergency care used to care for emergency psychiatric patients?

❑ The psychiatrist visits the patient in the emergency department.

❑ The Telephone consultation

❑ The psychiatric nurses visit the patient in the emergency department.

❑ Other…………………………………………………………………

**The assessment form of clarification of psychiatric emergency services policy**

**Explanation:** The form was developed to interview the head of the emergency department regarding the delivery of psychiatric emergency services. Please check ✓ into ❑

|  | **0**  Absent | **1**  Beginning | **2**  Basically effective | **3**  Mature plan | **4**  Advance | **5**  Role model |
| --- | --- | --- | --- | --- | --- | --- |
| 1. Improving the knowledge and skills of personnel in caring for emergency psychiatric patients. | ❑ no plans/projects for developing | ❑ There is a plan included in the hospital's service plan | ❑ There is a plan included in the hospital's service plan and implemented according to the plan | ❑ A plan is included in the hospital's service plan and implemented according to the plan, with a written outcome evaluation. | ❑ A plan is included in the hospital's service plan and implemented according to it; there is also a written evaluation, and the outcome evaluation is used for further development. | ❑ There is a plan included in the hospital's service plan and implemented according to the plan with a written evaluation. The outcome evaluation has been continually used for further development. |
| 2. Improving places & equipment for caring for emergency psychiatric patients within 48 hrs. | ❑ no plans/projects for developing | ❑ There is a plan included in the hospital's service plan | ❑ There is a plan included in the hospital's service plan and implemented according to the plan | ❑ A plan is included in the hospital's service plan and implemented according to the plan, with a written outcome evaluation. | ❑ A plan is included in the hospital's service plan and implemented according to it; there is also a written evaluation, and the outcome evaluation is used for further development. | ❑ There is a plan included in the hospital's service plan and implemented according to the plan with a written evaluation. The outcome evaluation has been continually used for further development. |
| 3 Preparing psychiatric medication and restraint devices for managing the behavior of emergency psychiatric patients. | ❑ no plans/projects for developing | ❑ There is a plan included in the hospital's service plan | ❑ There is a plan included in the hospital's service plan and implemented according to the plan | ❑ A plan is included in the hospital's service plan and implemented according to the plan, with a written outcome evaluation. | ❑ A plan is included in the hospital's service plan and implemented according to it; there is also a written evaluation, and the outcome evaluation is used for further development. | ❑ There is a plan included in the hospital's service plan and implemented according to the plan with a written evaluation. The outcome evaluation has been continually used for further development. |
| 4. Developing psychiatric emergency consultation systems | ❑ no plans/projects for developing | ❑ There is a plan included in the hospital's service plan | ❑ There is a plan included in the hospital's service plan and implemented according to the plan | ❑ A plan is included in the hospital's service plan and implemented according to the plan, with a written outcome evaluation. | ❑ A plan is included in the hospital's service plan and implemented according to it; there is also a written evaluation, and the outcome evaluation is used for further development. | ❑ There is a plan included in the hospital's service plan and implemented according to the plan with a written evaluation. The outcome evaluation has been continually used for further development. |
| 5. Developing clinical practice guidelines for emergency psychiatric care | ❑ no plans/projects for developing | ❑ There is a plan included in the hospital's service plan | ❑ There is a plan included in the hospital's service plan and implemented according to the plan | ❑ There is a plan included in the hospital's service plan and implemented according to the plan with a written outcome evaluation. | ❑ There is a plan included in the service plan of the hospital and implemented according to the plan; there is also a written evaluation, and the outcome evaluation is used for further development. | ❑ There is a plan included in the hospital's service plan and implemented according to the plan with a written evaluation, and the outcome evaluation has been continually used for further development. |
